# Dynamic functional hippocampal markers of residual depressive symptoms in euthymic bipolar disorder

**DOI:** 10.1002/brb3.3010

**Published:** 2023-04-16

**Authors:** Luigi F Saccaro, Julian Gaviria, Dimitri Van De Ville, Camille Piguet

**Affiliations:** ^1^ Faculty of Medicine, Psychiatry Department University of Geneva Geneva Switzerland; ^2^ Psychiatry Division Geneva University Hospital Geneva Switzerland; ^3^ Department of Basic Neurosciences University of Geneva Geneva Switzerland; ^4^ Swiss Center for Affective Sciences Campus Biotech Geneva Switzerland; ^5^ Faculty of Medicine, Department of Radiology and Medical Informatics University of Geneva Geneva Switzerland; ^6^ Neuro‐X Institute, School of Engineering Ecole Polytechnique Fédérale de Lausanne (EPFL) Geneva Switzerland; ^7^ Child and Adolescence Psychiatry Division Geneva University Hospital Geneva Switzerland

**Keywords:** biomarkers, bipolar disorder, functional brain imaging, hippocampus, magnetic resonance imaging

## Abstract

**Objectives:**

: Bipolar disorder (BD) is a severe, chronic, affective disorder characterized by recurrent switching between mood states, psychomotor and cognitive symptoms, which can linger in euthymic states as residual symptoms. Hippocampal alterations may play a key role in the neural processing of BD symptoms. However, its dynamic functional connectivity (dFC) remains unclear. Therefore, the present study explores hippocampal dFC in relation to BD symptoms.

**Methods:**

: We assessed hippocampus‐based dFC coactivation patterns (CAPs) on resting‐state fMRI data of 25 euthymic BD patients and 25 age‐ and sex‐matched healthy controls (HC).

**Results:**

: Bilateral hippocampal dFC with somatomotor networks (SMN) was reduced in BD, compared to HC, while at the same time dFC between the left hippocampus and midcingulo‐insular salience system (SN) was higher in BD. Correlational analysis between CAPs and clinical scores revealed that dFC between the bilateral hippocampus and the default‐like network (DMN) correlated with depression scores in BD. Furthermore, pathological hyperconnectivity between the default mode network (DMN) and SMN and the frontoparietal network (FPN) was modulated by the same depression scores in BD.

**Conclusions:**

: Overall, we observed alterations of large‐scale functional brain networks associated with decreased flexibility in cognitive control, salience detection, and emotion processing in BD. Additionally, the present study provides new insights on the neural architecture underlying a self‐centered perspective on the environment in BD patients. dFC markers may improve detection, treatment, and follow‐up of BD patients and of disabling residual depressive symptoms in particular.

## Graphical abstract

Graphical Abstract: Residual depressive symptoms in euthymic BD patients constitute one of the challenges of modern care of BD patients. By applying the cutting‐edge coactivation patterns (CAP) analysis to disentangle time‐varying fluctuations in resting‐state hippocampal dynamic functional connectivity (dFC), we highlight large‐scale functional brain networks associated with decreased flexibility in cognitive control, salience detection, and emotion processing in BD. Additionally, the present study provides new insights on lower hippocampal dFC with somatomotor networks (SMN) in BD, compared to HC, while at the same time dFC between the left hippocampus and midcingulo‐insular salience system (SN) was higher in BD. These abnormalities may underlie lower psychomotor activity due to disabling residual depressive symptoms in our euthymic BD patients, and a more self‐centered perspective of the environment, respectively. dFC markers may improve detection, treatment, and follow‐up of BD patients.

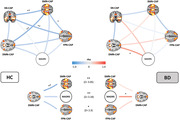

## 1 BACKGROUND

Bipolar disorder (BD) is a severe, chronic psychiatric affective disorder with a significant worldwide prevalence that can lead to suicide, poor quality of life as well as significant functional–occupational, educational, and interpersonal impairment (Blanthorn‐Hazell et al., 2018; Grande et al., 2016). It is characterized by recurrent switching between mood states (depressive, (hypo)manic, euthymic, and mixed), which differ in psychomotor, cognitive, and affective symptoms (Grande et al., 2016). According to the severity and extent of mood elevation, BD can be classified in type 1 or type 2 (Grande et al., 2016). Depressive symptoms often linger even in the euthymic state (Grande et al., 2016; Grover et al., 2020). These residual symptoms may include for instance psychomotor alterations, cognitive impairment, emotional dysregulation, and anxiety, with increased self‐centered thoughts and ruminations (Piguet et al., 2021; Saccaro et al., 2021). Identification and treatment of residual symptoms in euthymic BD patients is still insufficient, despite the fact that a consistent number of BD patients still struggle with disabling, pervasive and treatment‐resistant residual depressive symptoms, which constitute therefore a major challenge in the care of BD patients (Grande et al., 2016; Grover et al., 2020).

Despite the vast burden of BD (Grande et al., 2016), and notwithstanding significant efforts to identify effective interventions employing recent technologies (Saccaro et al., 2021), we are still lacking comprehensive pathophysiological insight, as well as neurobiological diagnostic or therapeutic markers of this complex disorder that might be multidimensional, including several variants with different neurobiological underpinnings. As a result, a high proportion of people struggling with BD do not benefit from adapted and currently available therapies (Grande et al., 2016). In an effort to disentangle the pathophysiological underpinnings of the disorder, alterations in several brain structures have been investigated in BD patients. In particular, alterations in emotion regulation networks have been consistently associated with BD (Sepede et al., 2015). Among the regions involved, the hippocampus seems to play a special role. Clinical neuroscience literature extensively supports the importance of the hippocampus in BD pathophysiology (Fateh et al., 2019; Haarman et al., 2016; Haukvik et al., 2020; Tu et al., 2017) and hippocampal volumetric and morphological abnormalities have been identified in lithium nonusers BD patients, who have smaller hippocampi compared to healthy controls (HC) (Haukvik et al., 2020). Furthermore, some effective BD pharmacological treatments appear to target hippocampal physiology and function (Palmos et al., 2021). While the hippocampus is primarily known for its role in memory and cognitive processes, including mnemonic reconstruction of scenes (Baldassano et al., 2016) and processing of external somatomotor information (involving the anterior hippocampus, in particular; Ezama et al., 2021), it is also pivotal in emotion regulation, being part of the prefrontal cortical–hippocampal–amygdala emotion processing circuit (Phillips & Swartz, 2014). These functions are interwoven, for instance, functional activity in the left hippocampus linked to autobiographical/episodic memory (Burgess et al., 2002) bears an emotional component, and might be the neural substrate for self‐centered thoughts and ruminations. While different hippocampal parcellations have been proposed, it is generally agreed that head–body–tail subdivisions exist (Genon et al., 2021; Plachti et al., 2019). Behavioral and functional profiling have highlighted what can be defined as an “emotion–cognition gradient” (Plachti et al., 2019; Robinson et al., 2015) along the anterior–posterior axis. The hippocampus thus reunites a set of cognitive representations and functions binding external somatomotor (or sensorimotor) information with internal affective features. Crucially, these functions overlap with the main domains affected by BD symptoms, that is, cognition, emotion, and somatomotor processing (Panchal et al., 2019; Phillips, 2006; Sepede et al., 2020; Sepede et al., 2012). The latter includes for instance motor agitation and hyperesthesia during mania, or the somatomotor slowing and dulling associated with depressive symptoms (Bowden, 2005). Interestingly, increasing evidence points to the involvement of somatomotor networks (SMN) in emotional and cognitive processing, with SMN having recently become a pivotal area of brain networks research in BD (Ellard et al., 2018; Kebets et al., 2019; Magioncalda et al., 2020; Ping et al., 2022; Saccaro et al., 2023; Tang et al, 2021; Wang et al., 2020; Zhu et al., 2022). Disrupted internetwork stationary functional connectivity (FC) has been shown in BD in the somatomotor network (SMN), default mode network (DMN), salience system (SN), and frontoparietal network (FPN). Most importantly, it has been suggested that abnormal stationary FC may be associated with BD symptoms (Ellard et al., 2018; Martino et al., 2020; Zhu et al., 2022), for instance, SMN FC was positively correlated with the Bech–Rafaelsen Mania Rating Scale in 18 BD patient (Zhu et al., 2022).

Results from studies on FC of the hippocampus as a pivotal region in BD remain inconclusive and heterogeneous. Previous works reported increased stationary FC between the hippocampus and frontal regions (Tu et al., 2017), or decreased hippocampus‐lingual gyrus stationary FC (Fateh et al., 2019). Similarly, task‐related hippocampal FC yielded contradictory results (Otten & Meeter, 2015). However, to the best of our knowledge, no study has investigated the relationship between hippocampal FC and BD symptoms. Notably, novel methodological approaches broaden our understanding of BD's impact on brain FC Recent efforts to characterize the time‐varying properties of BOLD signals using dynamic FC (dFC) (Bolton et al., 2020; Liu et al., 2018) allow us to better capture the dynamic of large‐scale networks typically impaired in BD. Nonetheless, little is known on dFC in BD yet. To the best of our knowledge, only one work compared dFC profiles with a sliding window method between 41 BD patients, 61 MDD (major depression disorder) patients, and 63 HC. The authors identified shared decreased DMN switching rates in depressed BD and MDD patients (Han et al., 2020), without investigating the association of these findings with psychiatric symptoms.

The present study leverages previous research and provides new insights on hippocampal resting‐state FC in BD by implementing coactivation patterns (CAPs) analysis, a dFC approach disentangling spatiotemporally overlapping interactions of a determined region of interest (i.e., hippocampus) with distinct brain regions (see (Bolton et al., 2020; Liu et al., 2018) for a full description of the CAPs method). This dFC analysis is particularly relevant to investigate the functional patterns of the hippocampus in BD. Unlike stationary FC, CAPs capture the dynamic features of time‐varying brain activity, characterizing the extensive functional connections of the hippocampus with multiple large‐scale brain networks (such as the FPN, SMN, and DMN) (Kim & Min, 2020). This is an outstanding feature when studying regions involved in multiple brain networks underlying multiple different symptoms domains, such as the hippocampus in BD.

Building on the hypothesis that disruption of hippocampal dFC may provide relevant markers of BD pathophysiology, here we aim to (1) capture hippocampal dFC patterns specific to BD patients, compared to HC and (2) investigate how interactions between these patterns is modulated by the severity of residual depressive symptoms in euthymic BD patients. Besides the potential aid in unraveling the complex pathophysiology of this severe disease, the goal of this project is to further identify neuroimaging markers of debilitating residual depressive BD symptoms.

## 2 METHODS

### 2.1 Participants

Twenty‐five euthymic BD patients satisfying DSM‐IV‐TR criteria were enrolled from the Mood Clinic of the Psychiatry Department of Geneva University Hospital according to a previously described protocol (Apazoglou et al., 2019). Briefly, trained psychologists interviewed them using the DIGS (Diagnostic Interview for Genetic Studies). Patients took part in the study following a 4‐week period of euthymic state (defined as Montgomery‐Åsberg Depression Rating Scale MADRS level <13, and Young Mania Rating Scale YMRS level <6) and on stable medication for 4 weeks. Twenty‐five age‐ and sex‐matched controls were recruited using local databases and web announcements. All participants provided written informed consent (ethical approval from Geneva University CER 13–081). Before the scanning session, all subjects completed clinical questionnaires detailed in the Supplementary Materials.

### 2.2 fMRI data acquisition

Whole‐brain MRI data were acquired with a 3T scanner (Siemens TIM Trio), and a 32 channels head‐coil, at the Brain & Behavior Laboratory, University of Geneva. The Blood Oxygenation Level Dependent (BOLD) contrast was measured using a T2*‐weighted echo‐planar sequence (EPI). Two hundred and fifty functional volumes of 36 axial slices each (TR/TE/flip angle = 2100 ms/30 ms/80°, FOV = 205 mm, resolution = 64 × 64, isotropic voxels of 3.2 mm^3^, distance factor 20%) were acquired. Furthermore, we collected a high‐resolution T1‐weighted anatomical image [TR/TI/TE/flip angle = 1900 ms/900 ms/2.27 ms/9°, FOV = 256 mm, resolution = 256 × 256, slice thickness = 1 mm^3^, 192 sagittal slices, phase encoding direction = anterior–posterior (AP)]. Our sequence included four dummy scans (∼9 s) at the beginning of the fMRI scanning. The subjects were instructed to lie awake, with normal breathing, and not think about anything in particular for 8 min.

### 2.3 fMRI data analysis

Neuroimaging resting‐state fMRI data were preprocessed using DPABI pipeline (www.restfmri.net), based on SPM12 toolkits (http://www.fil.ion.ucl.ac.uk/spm/software/spm12), as described in the Supplementary Materials. Head movement analysis did not reveal major head movement (head motion was equal to or lower than 2 mm translation or 2° rotation in any of the axes for all subjects). We scrubbed image volumes with frame‐wise displacement (FD) above 0.5 mm, and we discarded subjects with more than 25% of the scrubbed frames. Six motion parameters, white matter, and cerebrospinal fluid (CSF) signals were regressed out from the data as nuisance variables to reduce the impact nonneuronal BOLD fluctuations and motion.

To select our seed, in agreement with the tripartite model of the hippocampus (head–body–tail, anterior to posterior) (Genon et al., 2021; Plachti et al., 2019; Robinson et al., 2015), we adopted a robust hippocampal parcellation based on anatomical (Harvard Oxford atlas) and FC (task‐related and resting‐state) data, whose nodes were validated through meta‐analytic connectivity mapping (MACM) in two different databases (neurosynth and brainMap) (Plachti et al., 2019; Robinson et al., 2015). For better specificity, we selected as a seed the left and right anterior hippocampus, this subregion being the most involved in affective processing (Plachti et al., 2019; Robinson et al., 2015), a pivotal component of BD phenomenology, as described in the Introduction.

The TbCAPs software was employed for CAPs computation as described in detail elsewhere (Bolton et al., 2020; Rey et al., 2021) and in the Supplementary Materials. In brief, this toolbox extracts and Z‐scores the seed BOLD time‐series for each fMRI session, selecting the time points with the highest activity. Through K‐means clustering algorithm, the common pool of the retained whole‐brain volumes from all 50 subjects is agnostically classified into different clusters (CAPs), for which within‐cluster differences (defined as spatial similarity) were smaller than across‐cluster ones (more details about clustering are provided in Bolton et al., 2020). These CAPs were converted into spatial Z‐maps, so as to quantify the significance of their deviation from zero.

In total, five CAPs coactivating with the hippocampal seeds were identified, based on a data‐driven “consensus” procedure (Monti et al., 2003) that indicated *K* = 5 for each seed as the optimal number of distinct brain clusters in terms of replicability for this dataset. The fifth cluster did not show physiological patterns of brain activation and, after extensive verifications showing that it was consistently present in every methodological pipeline tested (see Supplementary Materials), it was discarded. The remaining four CAPs were analyzed.

We extracted for further analysis the temporal feature of CAPs’ occurrences, that is, defined as the sum of time points (frames) assigned to a given CAP among all the retained frames, across the entire timecourse. In other words, occurrences reflect how many times each subject's brain activity was in a certain spatial configuration indicated by the CAP when the hippocampus seed was active.

### 2.4 Statistical analyses

We implemented linear mixed models (LMM) (Pinheiro et al., 2022) in R (https://www.R‐project.org/) to compare the CAPs occurrences between groups (HC vs BD) controlling for sex, age, medication, and multiple clinical scores (further details are provided in the Supplementary Materials). The factor “group” referred to BD patients or controls, while “CAP” referred to CAPs’ occurrences. Additionally, “sex” and “age” and the clinical scores were modeled as fixed effects, and “subjects” included a random effect across subjects. The final LMM was:

Occurrences∼group×CAP+sex+age,random=∼1|subject.

The visual inspection of residual plots did not reveal any obvious deviations from homoscedasticity or normality. *p* Values were obtained by likelihood ratio tests (ANOVA) of the full model with the effect in question against the model without the effect in question. To dissect relationships and interactions between CAPs and BD symptoms, we ran Spearman's rank partial correlations (psych, 2021) between CAPs occurrences and the clinical scores. All results were adjusted for multiple comparisons using the FDR (false‐discovery rate) method.

## 3 RESULTS

### 3.1 Demographics and clinical scores

Twenty‐five BD patients (11 type 1, 13 type 2, 1 not otherwise specified) patients were included in the study, with 8 lifetime mood episodes on average (standard deviation, SD: 7). Supplementary Table S1 depicts the main demographic and clinical characteristics of our population. Of note, BD patients had significantly higher scores on the Montgomery–Åsberg Depression Rating Scale (MADRS), the affective lability scale (ALS), on a short version of the Ruminative Response Scale (RRS), and on the nonadaptive section of the emotion regulation questionnaire (CERQ), even in euthymic states. Further details on clinical scores and medication are provided in the Supplementary Materials.

### 3.2 CAPs identification

The four CAPs identified through the aforementioned steps were matched with well‐characterized main resting‐state networks based on the current literature (Uddin et al., 2019) as follows. A somatomotor‐visual CAP (SMN‐CAP, encompassing pericentral and visual areas, including also a part of the orbitofrontal cortex; i.e. Brodmann areas 11 and 12), a frontoparietal CAP (FPN‐CAP, also termed as the central executive network, CEN, or the executive control network, which involves the superior parietal lobule, the temporal complex, and frontal eyes fields), a default mode network CAP (DMN‐CAP), and a saliency‐network CAP (SN‐CAP, also termed salience network, midcingulo‐insular network, or cingulo‐opercular network, involving the anterior midcingulate cortex), bilateral insula as well as subcortical structures, including part of the ventral tegmental area (VTA) (Uddin et al., 2019).

### 3.3 Differences in temporal dynamics of hippocampal dynamic functional connectivity between BD patients and healthy controls

**FIGURE 1:**
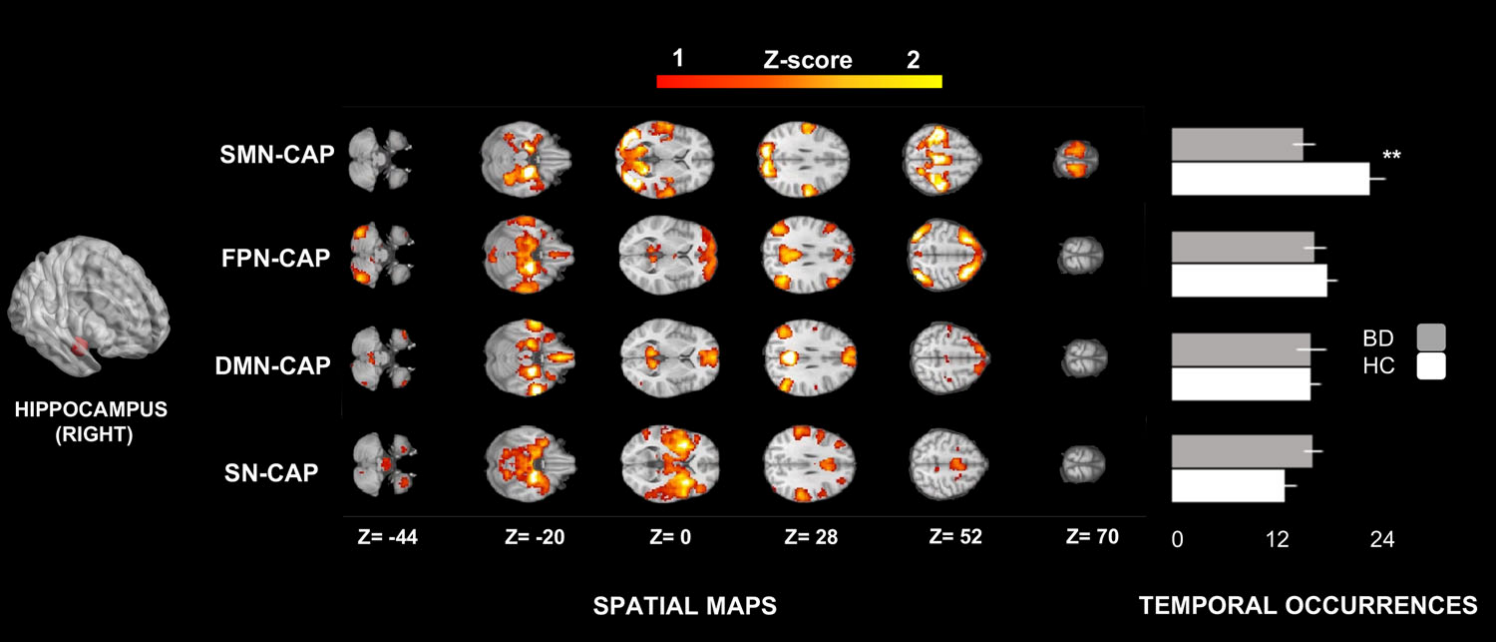
CAPs and temporal dynamics of the right hippocampus. We identified four CAPs using the right hippocampus as seed: a somatomotor‐visual CAP (SMN‐CAP), a frontoparietal CAP (FPN‐CAP), a default mode network CAP (DMN‐CAP) and a saliency‐network CAP (SN‐CAP). The occurrences of the SMN‐CAP were significantly lower in BD (bipolar disorder) patients than HC (healthy controls). Two stars indicate a significance level of *p* < .01; one star of *p* < .05, all adjusted for FDR (false‐discovery rate).

**FIGURE 2:**
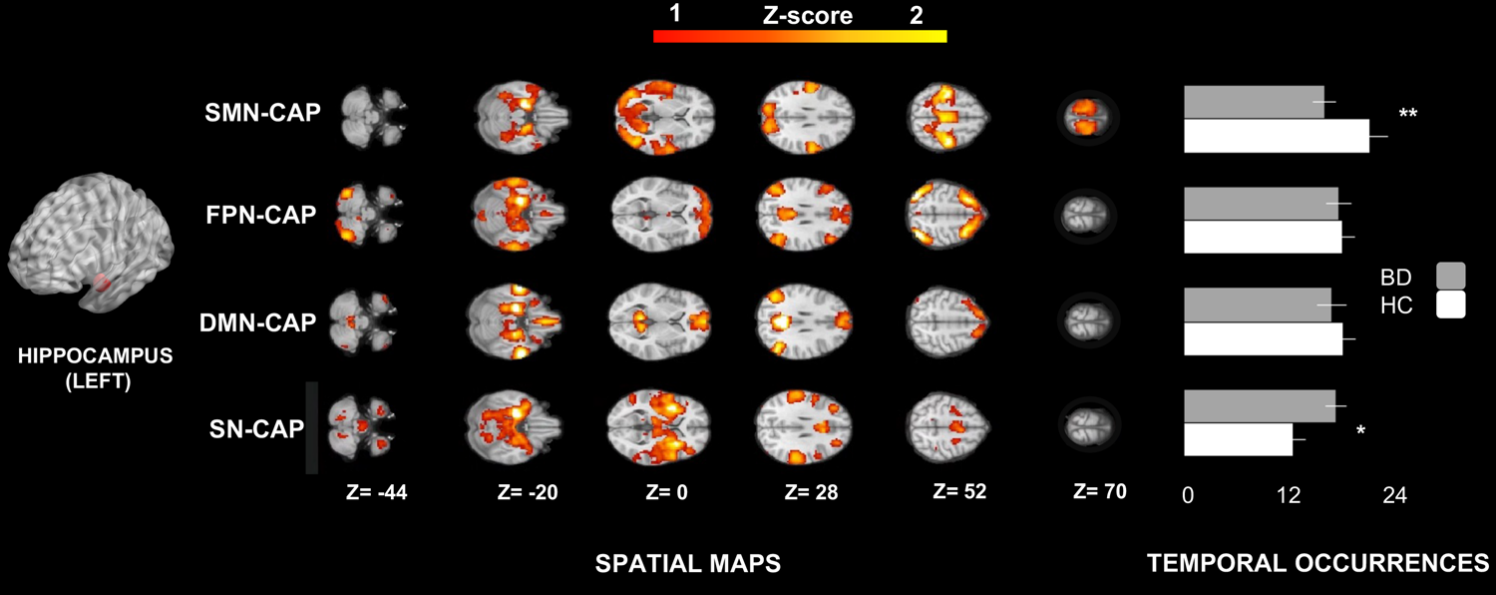
CAPs and temporal dynamics of the left hippocampus. We identified four CAPs using the left hippocampus as seed: a somatomotor‐visual CAP (SMN‐CAP), a frontoparietal CAP (FPN‐CAP), a default mode network CAP (DMN‐CAP) and a saliency‐network CAP (SN‐CAP). The occurrences of the SMN‐CAP were significantly lower in BD (bipolar disorder) patients than HC (healthy controls). The occurrences of the SN‐CAP were significantly higher in BD patients than HC. Two stars indicate a significance level of *p* < .01; one star of *p* < .05, all adjusted for FDR (false‐discovery rate).

The occurrences of the SMN‐CAP were significantly less frequent in BD patients than HC, no matter which seed was used. Indeed, this effect was present when using the right (β: –7.0, SE: 1.75, DF: 46, *t*: –3.98, *p*‐value_FDR_: .0002, Figure 1), the left (beta: –4.64, SE: 1.97, DF: 46, *t*‐ratio: –2.34, *p*‐value_FDR_: .02, Figure 2), or the bilateral hippocampus (beta: –3.65, SE:1.45, DF: 46, *t*‐ratio:‐2.51, *p*‐value_FDR_: .01, not shown) as seed.

Furthermore, the occurrences of the SN‐CAP were significantly higher in BD than HC using the left hippocampus as seed (beta: 4.36, SE: 1.97, DF: 46, *t*‐ratio: 2.20, *p*‐value_FDR_: .03, Figure 2).

There was no effect of either medication load or medication class on CAPs occurrences (see Supplementary Materials for further details).

### 3.4 Interactions between hippocampal dFC networks and clinical symptoms

**FIGURE 3:**
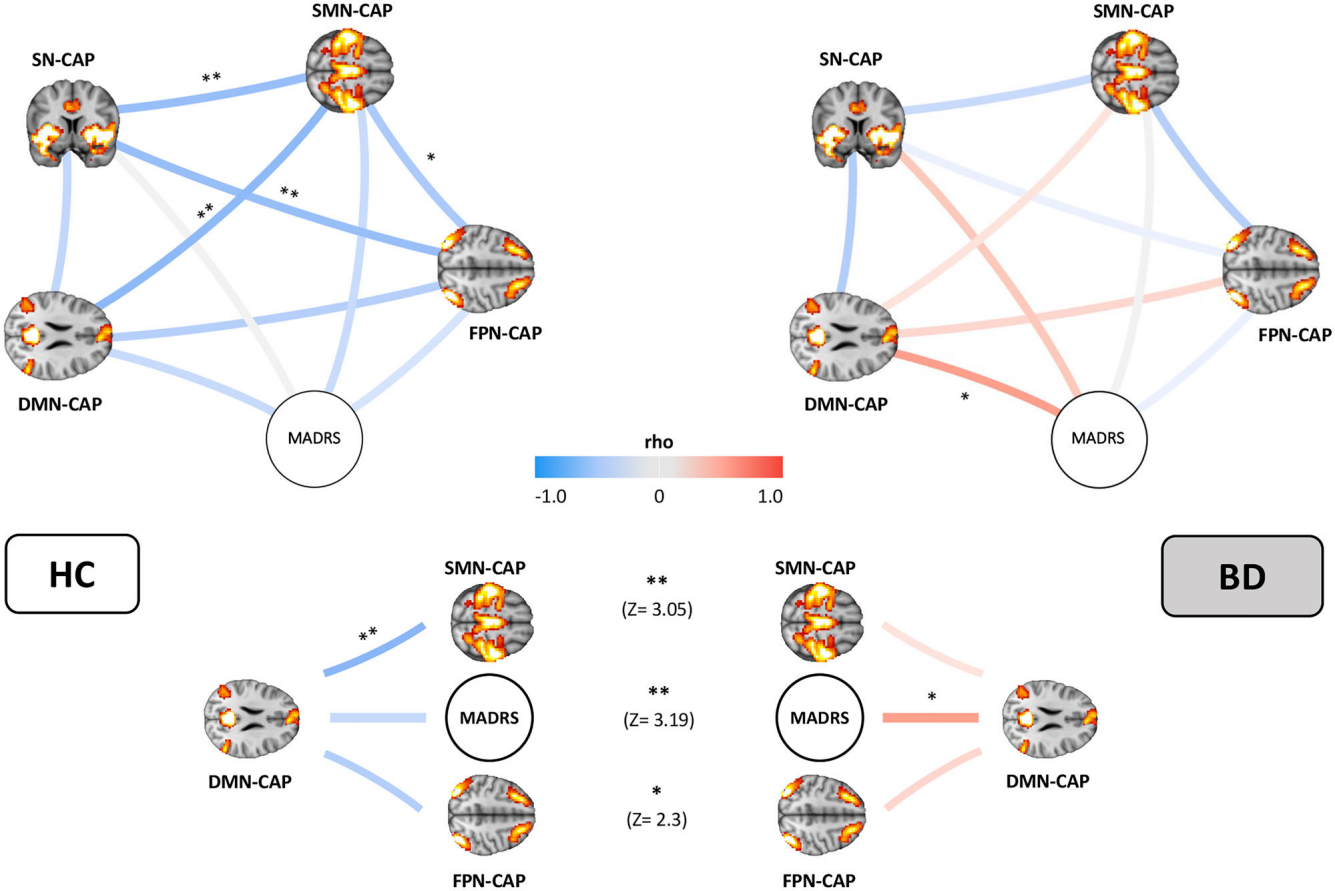
Functional interactions between CAPs occurrences and MADRS. The physiological interactions between the somatomotor‐visual CAP (SMN‐CAP), frontoparietal CAP (FPN‐CAP), default mode network CAP (DMN‐CAP), saliency‐network CAP (SN‐CAP), and a depression score (MADRS) are disrupted in bipolar patients (BD, right) compared to healthy controls (HC, left). Two stars indicate a significance level of *p* < .01; one star of *p* < .05, adjusted for FDR (false‐discovery rate).

We present here the partial correlation analysis among CAPS (seeded at the bilateral hippocampus) and including MADRS (Figure 3). MADRS was positively correlated with DMN occurrences (*p*‐value_FDR_< .05). In HC, we find a significant anticorrelation between DMN‐ and SMN‐CAPs, (*p*‐value_FDR_< .01), SN‐ and FPN‐CAPs (*p*‐value_FDR_< .01), SN and SMN‐CAPs (*p*‐value_FDR_< .01), and SMN‐ and FPN‐CAPs (*p*‐value_FDR_< .05). These interactions between different brain activity patterns (CAPs) were disrupted in BD. In fact, we found significant differences between BD and HC in the correlations of DMN‐CAP with (1) SMN‐CAP (*p*‐value_FDR_< .01), (2) MADRS (*p*‐value_FDR_< .01), and (3) FPN‐CAP (*p*‐value_FDR_< .05).

Overall in BD, we did not find any of the anticorrelations between CAPs occurrences found in HC, but rather a nonsignificant trend toward a positive correlation between DMN‐ and SMN‐CAPs (in contrast to the very significant anticorrelation between these networks in HC), and no correlation whatsoever between SN‐ and FPN‐CAPs (in contrast to the very significant anticorrelation between these networks in HC).

We confirm a similar disruption of the physiological correlations between brain networks in BD also when controlling for other clinical scores (Supplementary Figures S1–S3). In particular, when controlling for RRS, we report significant differences between BD and HC in the interactions of DMN‐CAP with SMN‐CAP (*p*‐value_FDR_< .05), and FPN‐CAP (*p*‐value_FDR_< .05), similarly to what we found when controlling for MADRS. Other secondary and nonsignificant findings on CAPs temporal dynamics and their interactions are detailed in the Supplementary Materials.

## 4 DISCUSSION

This is the first study on hippocampal dFC in BD disorder, employing CAPs analysis to disentangle time‐varying patterns of brain activity. The four CAPs that we identified through our data‐driven analysis of hippocampal dFC correspond to well‐characterized brain networks that physiologically connect with the hippocampus. As hypothesized, we found disrupted hippocampal dFC and large‐scale network interactions in BD compared with HC. Next, we discuss, first, different temporal dynamics (i.e., occurrences of each CAP) between BD patients and HC, and, second, the interactions between CAPs in BD and HC, also in relation to depressive symptoms.

### 4.1 Differences in temporal dynamics of hippocampal dynamic functional connectivity between BD patients and healthy controls

#### 4.1.1 dFC between the hippocampus and the somatomotor network

The hippocampus interacts with the SMN for the processing of external sensory information, in particular the anterior hippocampus (Ezama et al., 2021), and with the visual network in the context of mnemonic construction of scenes (Baldassano et al., 2016). We report lower dFC between both the right and the left hippocampus and the SMN‐CAP (involving somatomotor and visual networks) in BD during resting state. This finding is in agreement with accumulating results from different modalities of FC analysis on disruption of SMN in BD patients (Kebets et al., 2019; Martino et al., 2020; Rey et al., 2021; Saccaro et al., 2023; Tang et al., 2021). These suggest that SMN hypoconnectivity may predominate in more depressed states in association with reduced psychomotor activity, while SMN hyperconnectivity has been proposed to occur in (hypo)manic states (Martino et al., 2016) (when psychomotor agitation is more predominant), or in BD type 1 patients in general, in whom abnormal SMN intranetwork resting‐state FC has been shown to correlate with clinical symptoms and disrupted executive function (Zhu et al., 2022).

Here we also show significant SMN hypoconnectivity with the hippocampus in euthymic BD patients compared with HC. Considering that depressive symptoms often linger in euthymic BD patients (Grover et al., 2020) and that our euthymic BD patients had significantly higher depressive symptoms than HC, it is not unexpected that they could still present reduced psychomotor activity or increased vulnerability to depressive symptoms, and thus lower hippocampal‐SMN dFC than HC. This may explain the discrepancy between our findings and the aforementioned study (Martino et al., 2016), which did not find differences in SMN resting‐state FC of euthymic BD patients compared with controls. This discrepancy may also be due to the fact that we employed hippocampal dFC analyses (which, as discussed in the Background, may be more sensitive to temporally varying information) instead of whole‐brain stationary, resting‐state FC (Martino et al., 2016).

Thus, for the first time, we highlight the involvement of the hippocampus specifically in the aforementioned SMN deficits in BD. Considering the existing strong evidence on hippocampal structural abnormalities in BD (Haukvik et al., 2020) and its multiple roles in emotion, cognition, and sensory processing, the hippocampus appears as a crucial hub in BD symptomatology. In fact, besides BD psychomotor symptoms, also memory and cognitive dysfunction (domains traditionally related to the hippocampus) have been linked with SMN dysconnectivity in a transdiagnostic study including schizophrenic, schizoaffective, ADHD, and 40 BD patients (Kebets et al., 2019). Additionally, impaired SMN processing has been linked to emotion dysregulation (Kropf et al., 2019), which is a central BD symptom. In agreement with this, our results suggest that the well‐known hippocampal abnormalities in BD patients (Fateh et al., 2019; Haarman et al., 2016; Haukvik et al., 2020; Tu et al., 2017) might drive, at least partly, the disrupted SMN activity documented in BD (Rey et al., 2021; Martino et al., 2020; Kebets et al., 2019; Tang et al., 2021).

Thus, our findings confirm the SMN abnormalities in BD employing recent techniques of dFC analysis and pave the way to further research on the potential implications of hippocampal involvement in these alterations of SMN dFC.

#### 4.1.2 dFC between the hippocampus and the salience network

The SN includes both the “ventral attention network” and the “cingulo‐opercular network” (Uddin et al., 2019). While the ventral attention network is believed to mainly activate during exogenous salience identification (Uddin et al., 2019), the cingulo‐opercular network plays a broader role in the processing of personally relevant inputs (Uddin et al., 2019). Thus, our finding of higher dFC between the left hippocampus and the SN‐CAP (corresponding to the cingulo‐opercular network) in BD patients suggests a different assignment of saliency to external stimuli than in controls. Crucially, considering that the left hippocampus is particularly involved in context‐dependent autobiographical/episodic memory (Burgess et al., 2002), the fact that our results selectively implicate the left hippocampus may reflect a higher selective focus in BD patients on those features of the external world that are related to their personal autobiographical memory, with excessive focus on internal contents. Interestingly, our findings converge with previous evidence showing higher left‐hippocampal activity in BD during recognition of emotional images compared to HC and neutral images (Otten & Meeter, 2015; Whalley et al., 2009). Since also in this independent sample of BD patients only the left hippocampus was overly active (Kim & Min, 2020), it may be hypothesized that BD reactivity to the aforementioned emotional images (Kim & Min, 2020) was partly driven by increased self‐referential and autobiographical processing of external stimuli, related to activity lateralized to the left hippocampus (Burgess et al., 2002).

This is particularly interesting considering the aforementioned findings on reduced hippocampal dFC of our SMN‐CAP (which included visual networks), and the role of the hippocampus in the mnemonic construction of scenes (Baldassano et al., 2016). Taken together, our results may suggest that, during resting state, BD patients engage in environment exploration from a more self‐centered and autobiographical viewpoint, relying to a lower extent on the simple mnemonic reconstruction of their surroundings. In fact, higher dFC between the left‐hippocampal and SN in BD might underlie the first self‐centered mode of environment exploration (Burgess et al., 2002). On the other hand, lower dFC between the hippocampus and SMN in the same population may underlie lower recruitment of normal mnemonic reconstruction of the environment in BD (Baldassano et al., 2016).

Alternatively, aberrant and inappropriately high salience assignment (i.e., attributing disproportionate significance to irrelevant stimuli) has been proposed (Musa et al., 2021) as a marker of high risk for psychosis, a BD symptom, which might be mediated, at least partly, by hippocampal dysfunction and hyperconnectivity with the SN. This may represent a different explanation of the higher dFC between the hippocampus and the SN in our BD patients’ sample.

Finally, the finding of disrupted dFC in BD patients’ SN (including emotion processing circuits such as the insular cortex, amygdala, and ventral tegmental area) adds to the existing literature on abnormalities in emotion processing networks and the known emotion dysregulation in BD (Saccaro et al., 2021). However, further research is needed to test the reproducibility and validity of these speculations, since we did not find correlations between the SN and BD symptoms, contrary to previous evidence (Ellard et al., 2018).

These findings thus provide physiopathological insight and pave the way to further research on the association of SN abnormalities and BD symptoms.

### 4.2 Residual BD depressive symptoms modulate interactions between hippocampal dFC networks

MADRS scores were significantly higher in BD than HC, in agreement with the well‐known significant prevalence of euthymic BD patients that struggle with lingering depressive symptoms (Grover et al., 2020). We therefore explored the interaction between MADRS scores and hippocampal dFC network occurrences (Figure 3). Correlations between‐network occurrences and other clinical scores are described in the Supplementary Materials. In any case, independent of the clinical score used as a covariate of network temporal dynamics, the between‐network dFC analysis revealed diffuse and significant large‐scale network dysfunctions in BD compared with HC; i.e., dynamic relationships of networks anticoupling and coupling were significantly different in BD patients.

Broadly speaking, it has been hypothesized that the physiological anticorrelation between DMN, SN, and FPN is pivotal for cognitive control and adaptive mood regulation (Ellard et al., 2018). Thus, the disruption of the anticorrelation between the reported brain networks in BD may underpin deficient ability to switch neural resources from one network to the other, and disrupted communication between control (FPN) and brain networks engaged in internal, self‐referential thoughts (DMN), and emotion processing. Furthermore, this functional disruption may underlie biased self‐referential thoughts in BD (Apazoglou et al., 2019). Indeed, imbalanced dFC between networks involved in the regulation of external‐ or internal‐attention may underlie biases toward internally focused pathologic ruminations, at the cost of engaging with the environment during resting state, as shown for unipolar depression patients (Kaiser et al., 2015).

For these reasons, analysis of between‐networks dFC adds important information to the aforementioned results, as the interactions between large‐scale networks may differently shape the meaning of every single network's activity. In particular, while we described that the dFC between the bilateral hippocampus and the SMN‐CAP is reduced in BD, and we did not find differences between HC and BD in the occurrences of the FPN‐ and DMN‐CAPs, the between‐networks analyses showed that BD patients have significantly increased synchronization between the hippocampal‐DMN and the hippocampal‐SMN and ‐FPN dFC, compared to controls. In turn, hippocampal‐DMN dFC significantly correlates with depressive symptoms.

DMN engagement is associated with higher self‐referential processing, internal thoughts, and depressive symptoms in unipolar and bipolar patients (Piguet et al., 2021; Zhou et al., 2020). Our FPN‐CAP corresponds to the lateral‐FPN network, which, when connecting with the DMN components, has been implicated in internally focused attentional and cognitive control (Uddin et al., 2019). Anticorrelation between the DMN and FPN has been related to the capacity to flexibly redirecting attention away from internal processes, and toward external cues (Chou et al., 2022). Thus, we hypothesize that the significantly increased correlation between the DMN‐CAP and FNP‐CAP activity in BD represents a deficit in cognitive control switching, with additional involvement of internally oriented attentional processes in BD than in HC. Similarly, the significant reversal of the physiological anticorrelation between SMN and DMN in BD suggests a prevalently self‐centered interpretation of SMN information in BD. This is in agreement with previous findings (Piguet et al., 2021; Zhou et al., 2020) and with the aforementioned results on increased hippocampal‐SN dFC activity in BD, which is possibly associated with a more self‐centered environment exploration than in HC, as discussed above. This may seem counterintuitive considering our findings of reduced hippocampal‐SMN dFC in BD and the notion of overall reduced FC of SMN in BD presenting high depressive symptoms (Kebets et al., 2019; Martino et al., 2020; Martino et al., 2016; Rey et al., 2021; Tang et al., 2021). We hypothesize that, in BD patients showing high depressive symptoms, the reduced SMN FC with other networks is balanced by a hyperconnectivity of the SMN with the DMN, which has in fact been identified to be more predominant in depressed than euthymic BD patients (Liu et al., 2021). As mentioned, considering that depressive symptoms often linger in euthymic BD patients (Grover et al., 2020) and that our BD patients had significantly higher depressive symptoms than HC, it is not unexpected that they still presented higher DMN‐SMN dFC than HC.

Overall, these findings urge further research on the characterization of the balance between SMN and DMN networks as a potential BD marker. Most importantly, they suggest that BD patients traditionally defined as “euthymic” according to clinical scores may in fact still struggle with lingering depressive symptoms impacting their cerebral activity. Hippocampal dFC may provide useful instruments to clinicians to objectively quantify the impact of residual depressive symptoms on “euthymic” BD patients’ functioning, aiming at better patient stratification in research settings, therapeutical interventions, and prognostic assessments.

### 4.3 Conclusions, strengths, limitations, and perspectives

To conclude, we highlight dFC disruption within and between networks involved in externally (SMN) or internally (DMN) oriented attention, salience detection and emotion processing (SN), as well as goal‐directed regulation of these processes (FPN). Depression scores significantly interacted with disrupted DMN activity in BD patients in agreement with previous findings (Piguet et al., 2021; Zhou et al., 2020). Grounded in a conceptualization of BD symptoms arising from affect and cognitive control dysregulation neural circuitries linked to emotion and executive control functioning (Ellard et al., 2018), we postulate the increased cooccurrence between brain networks in BD as the neural substrate of decreased flexibility in adaptive mood regulation, in agreement with previous models (Ellard et al., 2018).

Concerning results on temporal dynamics of single hippocampal dFC networks, we hypothesize that during resting‐state BD patients engage in environment exploration from a more self‐centered and autobiographical viewpoint (higher left‐hippocampal‐SN dFC), relying to a lower extent on externally related information or simple mnemonic reconstruction of their surroundings (lower dFC between the hippocampus and the SMN).

The strengths of this study include the novelty of the dFC technique that we employed to study for the first time hippocampal dFC in BD patients age‐ and sex‐matched with HC, with significant advantages, compared to existing FC studies in BD, as detailed in the Background. Limitations of this study include small sample size, presence of comorbidities (detailed in Supplementary Table S1) and our study was not powered enough to run conclusive subanalyses (e.g., on effects of medication or BD type). However, we controlled our results for each of these variables without differences in significance (Supplementary Materials). Also, we cannot formally advance hypotheses on markers of BD state or subgroup differences. Similarly, analyses including clinical scores other than MADRS (Supplementary Materials) are limited by a lower statistical power due to missing data. Finally, we did not collect details on excluded participants.

Although further research is warranted, these findings may bear important diagnostic and prognostic implications. In particular, the specific pattern of disruption of the physiological dynamic organization of network correlations and anticorrelations of these interrelationships between large‐scale functional networks may represent a more complete BD neuroimaging marker than stationary FC markers or isolated intranetwork alterations, and it could potentially aid in differentiating bipolar from unipolar depression patients, who also shows a different pattern of large‐scale network dysfunction (Kaiser et al., 2015). Further, dFC markers are extremely promising as therapeutical markers thanks to their noninvasive nature and to the correlations with BD symptoms. For instance, they may represent useful therapeutical markers of mindfulness‐based cognitive therapy, which restored the normal anticorrelation between DMN and control networks in 22 BD patients, as a function of the ability to flexibly redirect attention from internal processes to the external environment (Chou et al., 2022). Alternatively, dFC markers may be used as tailored target outcomes guiding cultural–ecosocial interventions to improve emotion regulation and psychoeducation in BD, in agreement with current literature on an integrative therapeutical approach in psychiatry (Gómez‐Carrillo et al., 2023).

In addition, considering the link with depressive symptoms of the dFC alterations that we documented in euthymic BD patients, our results may speculatively help to stratify patients for research purposes. Namely, dFC markers may allow a more fine‐grained characterization of those BD patients that would otherwise be defined as purely “euthymic” according to clinical scores only. In this sense, further research is warranted on how hippocampal dFC may provide a much‐needed objective quantification of disabling and still ill‐treated residual depressive symptoms in “euthymic” BD patients, with the ultimate aim of improving treatment and follow‐up of people struggling with BD.

## CONFLICT OF INTEREST STATEMENT

The authors declare that they have no known competing financial interests or personal relationships that could have appeared to influence the work reported in this paper.

### FUNDING

This work was supported by the Swiss National Center of Competence in Research; “Synapsy: the Synaptic Basis of Mental Diseases” financed by the Swiss National Science Foundation [Grant Number 51NF40‐158776], as well as a grant of the Swiss National Science Foundation [Grant Number 32003B_156914].

### PEER REVIEW

The peer review history for this article is available at https://publons.com/publon/10.1002/brb3.3010.

## Supporting information

Supplementary Figure S1. Functional interactions between CAPs occurrences and RRS.
Supplementary Figure S2. Functional interactions between CAPs occurrences and CERQ.
Supplementary Figure S3. Functional interactions between CAPs occurrences and ALS.

## Data Availability

The data that support the findings of this study are available from the corresponding author upon reasonable request.
