# Supplementary material for: Dynamic functional hippocampal markers of residual depressive symptoms in euthymic bipolar disorder
Source: Brain Behav. 2023 Apr 16;13(6):e3010. doi: 10.1002/brb3.3010 (PMC10275545; doi:10.1002/brb3.3010)
Supplement: Supplementary file 1 — Supplementary Figure S1. Functional interactions between CAPs occurrences and RRS. Supplementary Figure S2. Functional interactions between CAPs occurrences and CERQ. Supplementary Figure S3. Functional interactions between CAPs occurrences and ALS. [file BRB3-13-e3010-s001.docx]

1. **SUPPLEMENTARY METHODS**
   1. ***Further description of clinical evaluation of the participants***

Before the scanning session, all subjects completed the Montgomery-Asberg Depression Rating Scale, the Young Mania Rating Scale (YMRS), the affective lability scale (ALS)^1^, the non-adaptive section of the emotion regulation questionnaire (CERQ)^2^, and a short version (10 items) of the Ruminative Response Scale (RRS)^3^. Other form of psychosis such as schizo-affective disorders were an exclusion criterion. the presence of an intellectual disability was excluded thanks to the Diagnostic Interview for Genetic Studies (DIGS, as described in the Methods), which includes a scoring for intellectual functioning, comorbidities, and attention. Additionally, we collected measures of working memory (such as mental calculation, forward and reverse digit span). All these assessments did not highlight intellectual disability in our participants.

- 1. ***Further description of fMRI data preprocessing***

We preprocessed the fMRI data using DPABI_V6.1 pipeline (www.restfmri.net), based on SPM12 toolkits (http://www.fil.ion.ucl.ac.uk/spm/software/spm12) with MATLAB 2018a (MathWorks, Inc., Natick, MA, USA). The preprocessing is described in detail elsewhere^4^, briefly the main steps included co-registration, realignment, signal regression, band-pass filtering, detrending, normalization to the standard Montreal Neurological Institute (MNI) EPI template, smoothing with a 4mm kernel. Additionally, we tested all possible combinations with multiple pipelines with or without slice time correction and/or global signal regression. The results presented are obtained from the pipeline without slice time correction nor global signal regression, but no major difference in the preprocessing results was highlighted between these different pipelines, and the main CAPs identified remained the same, as described below.

- 1. ***Basic principles of the CAPs analysis***

CAPs analysis disentangles different functional brain networks interacting with the seed region through a spatio-temporal frame-wise fMRI deconvolution. This approach conceptually agrees with the model of discrete transitions between brain meta-states^5^ and its physiological soundness is supported by multiple works, as described in^6^. Compared with stationary connectivity analyses, which neglect time-varying data, CAPs analysis captures neural activity fluctuations to cluster also spatio-temporally overlapping patterns of brain activation, which are thus not treated as mutually exclusive. Further, compared with other methods of dFC analysis, CAPs focus on single fMRI volumes at individual time points, instead of fMRI time courses, and are thus highly sensitive in identifying and analyzing brief, recurring, patterns of co-activation and their temporal variability^6,7^.

- 1. ***Fifth CAP exclusion and description of the different pipelines tested***

In total, five CAPs were identified, based on a data-driven “consensus” procedure^4^. The fifth CAP did not show physiological patterns of brain activation, and we hypothesized that it originated from clustering of artifacts. We will thus refer to it as the “artifacts-CAP”. To verify whether the artifacts-CAP was due to a certain methodological approach or rather to intrinsic noise in the data, we tested if different combinations of multiple pipelines had any effect on the artifacts-CAP, as follows.

Concerning the preprocessing, we tested all possible combinations of preprocessing pipelines with or without slice time correction and/or global signal regression, without changes in the artifacts-CAP (nor in the other four CAPs).

We hypothesized that the artifacts-CAP may be due to movement. However, there was no significant difference of frame-wise displacement (FD) power in the frames assigned to the five different CAPs (F-value: 1.594, p-value=0.2), nor in the artifacts-CAP in particular. Thus, movement does not seem related to the five CAPs that we identified.

We double-checked the wrapping to MNI through the quality control feature in DPABI, and verified that all subjects had excellent quality (5 on a scale from 1 to 5) of the normalization. We thus exclude that the artifacts-CAP is due to a problem in the normalization step.

While we choose to use 5 clusters based on a consensus algorithm, we explored also pipelines with 3,4,7, and 10 clusters (K=3,4,7, and 10). The artifacts-CAP was present in K=5,7, and 10, possibly suggesting that K=3,4 do not have enough clusters to efficiently isolate noise in a single CAP such as the artifacts-CAP. The SMN-CAP was present in all clustering approaches apart from K=10; the FPN-CAP was present in all clustering approaches apart from K=3; the DMN-CAP was present in all clustering approaches apart from K=7; the SN-CAP was present only in K=5 and K=10. Overall, these results suggest that the clusters that we identified are mostly stable and explain most of the signal of hippocampal dFC with low overlap. Crucially, only the solution with 5 clusters included all CAPs that corresponded to physiological, well-characterized networks.

Finally, inclusion of the artifacts-CAP in the statistical analysis did not change our results. Thus, since the artifacts-CAP did not show physiological patterns of brain activation, the aforementioned steps showed that its removal did not significantly impact our analyses, and we could verify that it was present notwithstanding different methodological approaches, we confirmed that it originates from clustering of noise and excluded it from further analyses. The presence of this artifacts-CAP probably increases the signal to noise ratio of the remaining CAPs, since it removes noise and artifacts from them, and the other CAPs show in fact very low noise.

- 1. ***Figures***

The seeds were visualized with the BrainNet Viewer (http://www.nitrc.org/projects/bnv/)^8^. The brain networks were visualized with Mango image processing software (Lancaster, Martinez; www.ric.uthscsa.edu/mango). Other graphs and figures were generated using Matlab©, Excel, PowerPoint, and R©.

1. **SUPPLEMENTARY RESULTS**
   1. ***Sub-analysis on medication***

The Spearman's rank correlation rho did not show significant correlations between CAPs occurrences and total psychotropic treatment load (zero, one, or more psychotropic medications) in BD patients (left hippocampus: p-value = 0.09, rho : -0.19; right hippocampus: p-value = 0.12, rho : -0.17; bilateral hippocampus: p-value = 0.5799, rho : -0.06).

To further explore the effect of medications, medication was grouped in four groups according to main neurotransmitter target: GABA (i.e. drugs that have a GABA enhancer or GABA agonist effect), 5HT/DA (serotonin and dopamine antagonists), GABA/5HT/DA (patients using both types of medication, or medication with both type of mechanism of action) and other (melatonin and natural remedies). Missing data were imputed as NA during the analysis. The effect of this classification of medication was then tested in a linear mixed effects model of the relationship between CAPs occurrences and medication class, including sex and age as covariates, by the following R-based formula syntax:

occurrences ~ medication * CAP + sex + age, random=~1|subject

where the independent variable “medication” refers to medication classes, “CAP” to each CAP, the fixed factors are sex and age of the subjects. We included a random effect for each subject. Visual inspection of residual plots did not reveal any obvious deviations from homoscedasticity or normality. P-values were obtained by likelihood ratio tests (ANOVA) of the full model with the effect in question against the model without the effect in question and did not show any interaction between CAPs occurrences and medication class (right hippocampus: p=0.57; left hippocampus: p=0.86, bilateral hippocampus: p=0.96). Healthy controls were not taking psychotropic treatments.

- 1. ***Sub-analysis on BD type***

A mixed model assessing the effect of BD type on CAPs occurrences and accounting for sex and age as covariates (“occurrences ~ BD type * CAP + sex + age + clinical score, random=~1|subject”) did not highlight any difference in occurrences of SMN and SN CAPs between BD type 1 and 2 (p>0.1 for all comparisons).

Of note, BD type 2 patients showed significantly higher hippocampal-DMN dFC than type 1 BD patients both when using the right (beta: -6.023, SE: 2.49, DF: 20, t-ratio: -2.4, p-value_FDR_: 0.02) or the left (beta: -7.2815, SE: 2.62, DF: 20, t-ratio: -2.7, p-value_FDR_: 0.011) hippocampus as seed. Considering that BD type 2 patients showed a non-significant trend towards higher MADRS scores, this is in agreement with the significant positive correlation between MADRS scores and hippocampal-DMN dFC detailed in the manuscript. However, our analysis was not powered enough to compare BD type 1 and 2, due to small sample size (11 type 1, 13 type 2 BD patients).

- 1. ***Non-significant findings on temporal dynamics of hippocampal dFC***

dFC between the hippocampus and the FPN-CAP was not different between BD and HC, suggesting that hippocampal networks of attention and control are not impacted by BD diagnosis, at least in the euthymic state. This is in agreement with the rest of our results and with existing literature^9^, suggesting that the object of attention might be different in BD patients (who likely show increased focus on internal and self-related thoughts), not the attention processes themselves, as detailed in the Discussion.

An apparently counter-intuitive finding is the absence of difference in hippocampal dFC with the DMN-CAP between BD and HC. Since, overall, our findings suggest increased self-focused and internal processing in BD, we would expect the DMN-CAP occurrences to be increased in BD compared with HC. However, this is in agreement with existing findings from a systematic review on euthymic BD^10^, and DMN abnormalities have mostly been highlighted in unipolar and bipolar depression^11^, while our BD patients were globally euthymic despite sub-threshold depressive symptoms highlighted by higher MADRS scores than HC. Thus, it is possible that euthymic BD patients do not show clear DMN dFC abnormalities, or that these are subtle, and our study was not powered enough to detect them. Further, we describe in the Manuscript numerous significant differences between BD and HC in the correlations between DMN and MADRS or other brain networks. This might suggest that there are indeed between-network DMN abnormalities in our sample, but that the specific, intra-network dFC between the hippocampus and the DMN is not affected by BD diagnosis.

- 1. ***Interactions between CAPs’ occurrences when controlling for different clinical scores***

As mentioned (**Supplementary** **Table 1**), BD patients had significantly higher scores on the affective lability scale (ALS), on a short version of the Ruminative Response Scale (RRS), and on the non-adaptive section of the emotion regulation questionnaire (CERQ). We therefore investigated how interactions between CAPs occurrences varied when controlling for each of these scores, besides MADRS scores (described above).

It is interesting how CAPs occurrences differentially related to clinical scores. SMN-CAP (rho=0.50) and FPN-CAP (rho=0.51) were positively (but non-significantly) correlated with RRS rumination scores in BD patients (**Supplementary Figure** **1**). These correlations (SMN, FPN with RRS scores) are not significant probably due to lack of power (i.e., small sample due to missing data, N=18). There is a wealth of research showing an association between rumination tendencies and a deficit in cognitive control (FPN) suggesting that a dysfunctional cognitive processing with overactive internal-focus may disrupt control and transitions from internal to external focus in BD^12,13^. On the other hand, to the best of our knowledge the association between rumination and SMN is less studied.


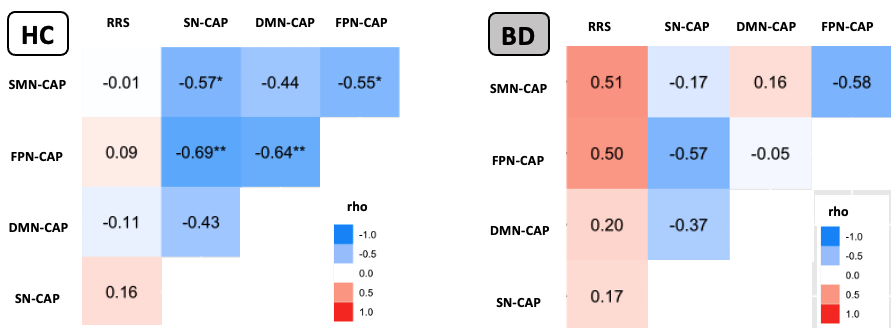


**Supplementary Figure** **1. Functional interactions between CAPs occurrences and RRS.** The physiological interactions between the somatomotor-visual CAP (SMN-CAP), frontoparietal CAP (FPN-CAP), default mode network CAP (DMN-CAP), saliency-network CAP (SN-CAP), and the rumination score (RRS) are disrupted in bipolar patients (BD, right) compared to healthy controls (HC, left). Two stars indicate a significance level of p<0.01; one star of p<0.05, adjusted for FDR, false discovery rate.

We identified other similar trends when controlling for CERQ (**Supplementary Figure** **2**) or ALS (**Supplementary Figure** **3**), but, likely due to missing data leading to lack of power, these results are mostly not significant. It is remarkable, however, that the positive correlation between DMN and SMN that we observed when controlling for MADRS, became significant (rho=0.67, p-value_FDR_<0.05) when controlling for ALS instead (**Supplementary Figure** **3**). While it could thus be hypothesized that a component of the abnormal hyperconnectivity between DMN and SMN is due to affective lability and not only to residual depressive symptoms, further research on larger sample is needed to investigate these relationships.


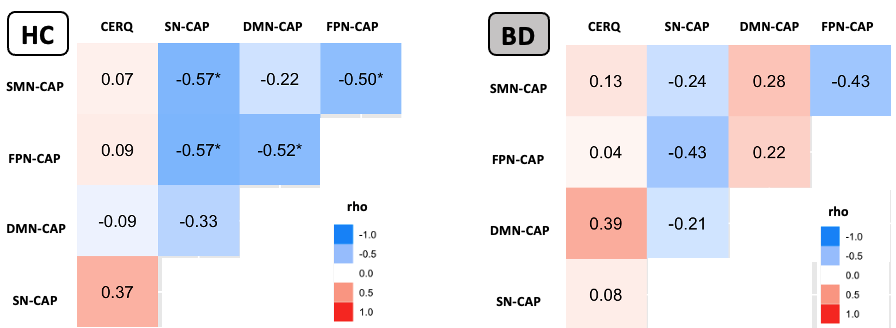


**Supplementary Figure** **2. Functional interactions between CAPs occurrences and CERQ.** The physiological interactions between the somatomotor-visual CAP (SMN-CAP), frontoparietal CAP (FPN-CAP), default mode network CAP (DMN-CAP), saliency-network CAP (SN-CAP), and the non-adaptive emotional regulation score (CERQ) are disrupted in bipolar patients (BD, right) compared to healthy controls (HC, left). Two stars indicate a significance level of p<0.01; one star of p<0.05, adjusted for FDR, false discovery rate.

**
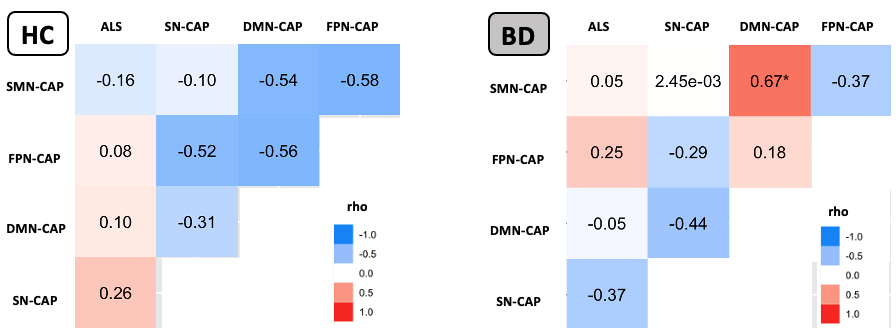
**

**Supplementary Figure** **3. Functional interactions between CAPs occurrences and ALS.** The physiological interactions between the somatomotor-visual CAP (SMN-CAP), frontoparietal CAP (FPN-CAP), default mode network CAP (DMN-CAP), saliency-network CAP (SN-CAP), and the affective lability score (ALS) are disrupted in bipolar patients (BD, right) compared to healthy controls (HC, left). Two stars indicate a significance level of p<0.01; one star of p<0.05, adjusted for FDR, false discovery rate.

We did not run sub-analysis including the YMRS due to missing data and no significant difference in BD/HC results controlling for this score.

**SUPPLEMENTARY TABLE AND TABLE LEGEND**

|  | **BD patients (N=25)** | **Controls (N=25)** | **p-value** |
| --- | --- | --- | --- |
| ***Demographics*** |  |  |  |
| Age: mean (SD) | 31.7 (11.4) | 30 (10.8) | 0.6 |
| Females/males | 12/13 | 12/13 | 0.8 |
| Education, mean (SD) | 14 (3.5) | 14.4 (3.2) | 0.6 |
| ***Clinical*** |  |  |  |
| BD type 1/2 | 11/13 | NA | NA |
| ALS: mean (SD) | 1.1 (0.7) | 0.4 (0.3) | 0.00076 |
| MADRS: mean (SD) | 3.8 (3.4) | 1.2 (1.8) | 0.002 |
| YMRS: mean (SD) | 0.1 (0.4) | 0 (0) | 0.6 |
| CERQ: mean (SD) | 53.6 (28) | 37.3 (20) | 0.01 |
| RRS: mean (SD) | 24.1 (5.8) | 18.5 (4) | 0.01 |
| ***Disease severity*** |  |  |  |
| Number of lifetime mood episodes: mean (SD) | 8 (7) | NA | NA |
| Disease duration, mean (SD) | 13 (9.3) | NA | NA |
| Hospitalizations: mean (SD) | 3.8 (3.7) | NA | NA |
| ***Comorbidites*** |  |  |  |
| PTSD | 2 | 0 |  |
| TOC | 2 | 0 |  |
| ADHD | 9 | 0 |  |
| Anxiety disorders | 9 | 0 |  |

**Supplementary Table 1. Participants’ demographic and clinical characteristics.** The table recapitulates main demographic and clinical characteristics of BD patients and HC. Affective lability scale (ALS), bipolar disorder (BD), non-adaptive subscore of the emotion regulation questionnaire (CERQ), Montgomery–Åsberg Depression Rating Scale (MADRS), not applicable (NA), healthy controls (HC), Ruminative Response Scale (RRS), standard deviation (SD).

1. **SUPPLEMENTARY REFERENCES**

1. Harvey PD, Greenberg BR, Serper MR. The affective lability scales: Development, reliability, and validity. *J Clin Psychol*. 1989;45(5):786-793. doi:10.1002/1097-4679(198909)45:5<786::AID-JCLP2270450515>3.0.CO;2-P

2. Garnefski N, Kraaij V, Spinhoven P. Negative life events, cognitive emotion regulation and emotional problems. *Pers Individ Dif*. 2001;30(8):1311-1327. doi:https://doi.org/10.1016/S0191-8869(00)00113-6

3. Treynor W, Gonzalez R, Nolen-Hoeksema S. Ruminative reconsiderd: A psychometric analysis. *Cognit Ther Res*. 2003;27(3):247-259. https://link.springer.com/content/pdf/10.1023/A:1023910315561.pdf%0Ahttps://journals-scholarsportal-info.ezproxy.library.yorku.ca/pdf/01475916/v27i0003/247_rrapa.xml.

4. Yan C-G, Wang X-D, Zuo X-N, Zang Y-F. DPABI: Data Processing & Analysis for (Resting-State) Brain Imaging. *Neuroinformatics*. 2016;14(3):339-351. doi:10.1007/s12021-016-9299-4

5. Vidaurre D, Smith SM, Woolrich MW. Brain network dynamics are hierarchically organized in time. *Proc Natl Acad Sci U S A*. 2017;114(48):12827-12832. doi:10.1073/pnas.1705120114

6. Liu X, Zhang N, Chang C, Duyn JH. Co-activation patterns in resting-state fMRI signals. *Neuroimage*. 2018;180(February):485-494. doi:10.1016/j.neuroimage.2018.01.041

7. Bolton TAW, Tuleasca C, Wotruba D, et al. TbCAPs: A toolbox for co-activation pattern analysis. *Neuroimage*. 2020;211:116621. doi:https://doi.org/10.1016/j.neuroimage.2020.116621

8. Xia M, Wang J, He Y. BrainNet Viewer: A Network Visualization Tool for Human Brain Connectomics. *PLoS One*. 2013;8(7):1-15. doi:10.1371/journal.pone.0068910

9. Chou T, Dougherty DD, Nierenberg AA, Deckersbach T. Restoration of default mode network and task positive network anti-correlation associated with mindfulness-based cognitive therapy for bipolar disorder. *Psychiatry Res - Neuroimaging*. 2022;319(July 2021):111419. doi:10.1016/j.pscychresns.2021.111419

10. Syan SK, Smith M, Frey BN, et al. Resting-state functional connectivity in individuals with bipolar disorder during clinical remission: A systematic review. *J Psychiatry Neurosci*. 2018;43(5):298-316. doi:10.1503/jpn.170175

11. Piguet C, Karahanoğlu FI, Saccaro LF, Van De Ville D, Vuilleumier P. Mood disorders disrupt the functional dynamics, not spatial organization of brain resting state networks. *NeuroImage Clin*. 2021;32. doi:10.1016/j.nicl.2021.102833

12. Apazoglou K, Küng AL, Cordera P, et al. Rumination related activity in brain networks mediating attentional switching in euthymic bipolar patients. *Int J Bipolar Disord*. 2019;7(1). doi:10.1186/s40345-018-0137-5

13. Marchetti I, Koster EHW, Klinger E, Alloy LB. Spontaneous Thought and Vulnerability to Mood Disorders: The Dark Side of the Wandering Mind. *Clin Psychol Sci a J Assoc Psychol Sci*. 2016;4(5):835-857. doi:10.1177/2167702615622383

14. Liu M, Wang Y, Zhang A, et al. Altered dynamic functional connectivity across mood states in bipolar disorder. *Brain Res*. 2021;1750(May 2020):147143. doi:10.1016/j.brainres.2020.147143
